# Cancer Survivors’ Experience With Telehealth: A Systematic Review and Thematic Synthesis

**DOI:** 10.2196/jmir.6575

**Published:** 2017-01-09

**Authors:** Anna Cox, Grace Lucas, Afrodita Marcu, Marianne Piano, Wendy Grosvenor, Freda Mold, Roma Maguire, Emma Ream

**Affiliations:** ^1^ School of Health Sciences Faculty of Health and Medical Sciences University of Surrey Guildford United Kingdom

**Keywords:** neoplasms, telemedicine, systematic review, survival, patient satisfaction, patient preference

## Abstract

**Background:**

Net survival rates of cancer are increasing worldwide, placing a strain on health service provision. There is a drive to transfer the care of cancer survivors—individuals living with and beyond cancer—to the community and encourage them to play an active role in their own care. Telehealth, the use of technology in remote exchange of data and communication between patients and health care professionals (HCPs), is an important contributor to this evolving model of care. Telehealth interventions are “complex,” and understanding patient experiences of them is important in evaluating their impact. However, a wider view of patient experience is lacking as qualitative studies detailing cancer survivor engagement with telehealth are yet to be synthesized.

**Objective:**

To systematically identify, appraise, and synthesize qualitative research evidence on the experiences of adult cancer survivors participating in telehealth interventions, to characterize the patient experience of telehealth interventions for this group.

**Methods:**

Medline (PubMed), PsychINFO, Cumulative Index for Nursing and Allied Health Professionals (CINAHL), Embase, and Cochrane Central Register of Controlled Trials were searched on August 14, 2015, and March 8, 2016, for English-language papers published between 2006 and 2016. Inclusion criteria were as follows: adult cancer survivors aged 18 years and over, cancer diagnosis, experience of participating in a telehealth intervention (defined as remote communication or remote monitoring with an HCP delivered by telephone, Internet, or hand-held or mobile technology), and reporting qualitative data including verbatim quotes. An adapted Critical Appraisal Skill Programme (CASP) checklist for qualitative research was used to assess paper quality. The results section of each included article was coded line by line, and all papers underwent inductive analysis, involving comparison, reexamination, and grouping of codes to develop descriptive themes. Analytical themes were developed through an iterative process of reflection on, and interpretation of, the descriptive themes within and across studies.

**Results:**

Across the 22 included papers, 3 analytical themes emerged, each with 3 descriptive subthemes: (1) influence of telehealth on the disrupted lives of cancer survivors (convenience, independence, and burden); (2) personalized care across physical distance (time, space, and the human factor); and (3) remote reassurance—a safety net of health care professional connection (active connection, passive connection, and slipping through the net). Telehealth interventions represent a convenient approach, which can potentially minimize treatment burden and disruption to cancer survivors’ lives. Telehealth interventions can facilitate an experience of personalized care and reassurance for those living with and beyond cancer; however, it is important to consider individual factors when tailoring interventions to ensure engagement promotes benefit rather than burden.

**Conclusions:**

Telehealth interventions can provide cancer survivors with independence and reassurance. Future telehealth interventions need to be developed iteratively in collaboration with a broad range of cancer survivors to maximize engagement and benefit.

## Introduction

The term “cancer survivor” is used to encompass all individuals living with cancer “from the time of diagnosis and for the balance of life” [1]. Rates of cancer survival are considered a key metric for cancer control [2]. There are 2.5 million cancer survivors in the United Kingdom and this is predicted to increase to 4 million by 2030, in line with the increase in both cancer incidence and net survival rates identified for many cancer types worldwide between 1995 and 2009 [3]. Lifetime risk of cancer now varies between 33% in Australia before 75 years [4] to over 50% in the United Kingdom for those born after 1960 [5]. Variation in European cancer survival rates is associated with levels of health services funding and organization [6,7], with such relationships also being observed in other countries worldwide [8]. This exponential rise in cancer survivors is met by finite resources, thereby placing considerable strain on cancer service provision. Consequently, alternative approaches to service delivery and provision of supportive care are needed and are driving technological innovation in health care [9].

The effort to develop and implement technological innovations to support cancer survivorship is a global one, reflecting the drive to transfer the care of cancer survivors from hospital to community settings [10] and encourage them (and their families or caregivers) to play an active role in managing their care [11]. This evolving model of cancer care has led researchers to investigate use of telehealth in health care delivery. Telehealth refers to the use of technology to provide remote personalized health care to patients [12,13] which allows exchange of data and communication between patients and health care professionals (HCPs) [14]. Examples of initiatives in active development include the UK-based eRAPID remote symptom reporting system [15], a Web-based exercise program in the Netherlands [16] and rural chemotherapy administration under guidance from centrally-based oncologists in Australia [17].

However, little is known about cancer survivors’ engagement with, and acceptance of, cancer telehealth interventions, and their lived experience of being remotely monitored—often the focus is on intervention outcomes. Of recent reviews, 3 sought to synthesize trial findings from studies reporting outcomes from interventions tested with cancer survivors in a supportive capacity [18,19] and in the delivery of follow-up [20]. They appraised benefits in terms of quality of life and management of symptoms (including pain, depression, anxiety, fatigue, and sexual dysfunction) using patient-reported outcome measures, but found the benefit of telehealth to vary between studies. One review of supportive telehealth interventions [18] was inconclusive regarding their efficacy in reducing depression (only 4 of 9 studies focusing on depression reported significant effects) but did suggest benefits in terms of reducing pain (of the 3 studies on pain control, 2 reported significant effects). Another review of supportive telehealth interventions [19] found that 9 of 20 studies indicated a significant improvement in at least one psychosocial outcome measure. However, only one of these found that this improvement was sustained at the end of the follow-up period [21]. The review appraising research addressing remote follow-up [20] concluded that this form of telehealth neither significantly decreased psychological distress, nor enhanced quality of life of cancer survivors. Only 2 studies reviewed reported significant improvements in quality of life or fatigue levels. This suggests that the current evidence on telehealth effectiveness is relatively mixed and that the type of telehealth intervention employed may impact final outcomes.

Telehealth interventions are “complex,” comprising many components, and can be time consuming and expensive to develop and test. Medical Research Council guidance on developing and evaluating complex interventions highlights the importance of qualitative research for developing the theoretical understanding of complex interventions’ impact and processes of action [22]. The systematic reviews discussed above synthesized solely results from studies reporting patient-reported outcomes. However, they did not incorporate elements pertaining to patients’ needs for, or experiences of, or engagement with telehealth. Yet, these are important considerations for successful uptake of telehealth interventions. Systematic reviews conducted to date have primarily enabled consideration of whether telehealth offers benefit to cancer survivors [18-20], but a qualitative synthesis of the cancer survivor’s experience of telehealth will enable consideration of how and why cancer survivors experience any benefit, or not.

The aim of this review therefore was to systematically identify, appraise, and synthesize qualitative research evidence on the experience of adult cancer survivors who have engaged with telehealth intervention(s) and provide a fine-grained understanding of users’ perspectives. The intent was to enhance characterization of the impact of telehealth interventions upon the experience of cancer survivorship and identify potential steps to improve engagement of cancer survivors with telehealth.

## Methods

The reporting of this qualitative synthesis follows the Enhancing Transparency in Reporting the Synthesis of Qualitative Research (ENTREQ) guidelines [23].

### Search Strategy

A comprehensive search strategy was developed to identify all the studies relevant to our research question. The search strategy was developed for Medline (PubMed), then adapted and applied to PsychINFO, Cumulative Index for Nursing and Allied Health Professionals (CINAHL), Embase, and the Cochrane Central Register of Controlled Trials. These databases were chosen to encompass nursing, medicine, social sciences, and psychology. To retrieve other relevant publications, the reference lists of selected publications were hand searched and articles considered against the eligibility criteria. Nonresearch publications and “gray” literature were excluded. The search was conducted on August 14, 2015, and updated on March 8, 2016. Search results were uploaded and stored using Endnote version 7.4 (Clarivate Analytics). Duplications of studies were removed.

Search terms were split into 3 categories: cancer survivors (population), eHealth (intervention), and survivor experience (outcome). Each category included medical subject headings (MeSH) and keywords using trunctation (*) within title or abstract fields (see Multimedia Appendix 1 for full Medline search strategy). The search terms were informed by previous systematic reviews of eHealth [24,25] and database thesauri. Broad search terms were used for eHealth, rather than the more restrictive term “telehealth,” to ensure that all relevant interventions were captured. Boolean terms “OR” and “AND” were used to combine searches within and between categories respectively. The search was restricted to papers published in English between 2006 and 2016 to encompass recent papers of the last decade for relevancy. Database searches are less successful at identifying qualitative studies, and abstracts of studies reporting qualitative data are variable in content, not always indicating the research method [26]. Consequently, the initial search was not limited by research design; papers which incorporated qualitative data were identified at the stage of assessing full text articles for eligibility.

### Inclusion and Exclusion Criteria

Textboxes 1 and 2 present the papers eligible for inclusion in and exclusion from the study.

**Inclusion criteria for the study.**

- Original articles in English published in the period of January 1, 2006, to March 8, 2016
- Papers reporting on adults (over 18 years) who had received a diagnosis of cancer, regardless of gender, tumor type, or comorbidities
- Papers reporting on participants who had experienced a telehealth intervention, which enabled remote communication or remote monitoring with health care professionals (HCPs) (the main component of the intervention was delivered by telephone, using the Internet, or using hand-held or mobile technology)
- Papers reporting qualitative data—including verbatim quotes—on cancer survivors’ experience of using a telehealth intervention

**Exclusion criteria for the study.**

- Broader experience of eHealth—did not provide remote communication or monitoring with health care professionals (HCPs) (eg, chat rooms, social media, remote peer support)
- Data collected during development of an intervention based on the user’s expectations rather than experience
- Experience of carers only
- Experience of users with conditions other than cancer
- Gray literature or reviews
- Qualitative data that did not include verbatim quotes

### Screening and Data Extraction

A 2-stage screening process was conducted. In stage one, 3 reviewers (first author and 2 members of the research team) screened all identified titles and abstracts that were potentially eligible for inclusion irrespective of research methodology. Full papers were then obtained and potentially eligible studies were assessed for inclusion independently by at least two of the 5 members of the review team (AC, AM, WK, FM, RM); at this stage papers that did not incorporate qualitative data were excluded. Uncertainties around paper inclusion were resolved by the final member of the review team (last author) if necessary.

All members of the research team independently extracted data for each paper using a data extraction form devised by the team; data from each paper were extracted twice by 2 separate members. All text from the papers labelled as “results” or “findings” was extracted electronically and entered into Nvivo 10, a qualitative data analysis computer software package (QSR International). Data extraction forms were compared across reviewers for each paper to ensure accuracy and comprehensiveness of data extraction.

### Quality Assessment

The review team adapted the Critical Appraisal Skill Programme (CASP) checklist for qualitative research [27] to include assessment of information power, a concept proposed by Malterud et al [28] as an alternative to saturation in qualitative research. Information power refers to how researchers can achieve adequate sample size in qualitative studies by having a clearly defined aim, a specific sample, a theoretical approach, high quality dialogue, and clear analytic strategy. The adapted tool was piloted on a subsample of studies (n=12) by 6 members of the review team (AC, AM, FM, ER, RM, WG). Following minor amendments, the tool was used independently by 2 members of the review team to assess the remaining studies. All studies fulfilling the eligibility criteria were assessed with the adapted checklist comprising: research design, sampling strategy, analysis, presentation and interpretation of findings, reflexivity, ethical considerations, relevance, and transferability. The decision was made to include all studies in the analysis, however, less emphasis was given to studies assessed by the checklist as relatively lacking in rigor.

### Thematic Synthesis

The findings of primary research studies were synthesized using methods proposed by Thomas and Harden [29]. These methods aim to achieve a high level of analysis and integration via 3 stages of synthesis: (1) Line-by-line coding of the results section of each paper, (2) development of descriptive themes which remain close to the themes from the primary research, and (3) development of analytical themes, which go beyond the primary research findings and generate a higher level of conceptual understanding.

Of the review team, 2 members (AC and GL) coded the results section of each included article line by line and developed descriptive themes through inductive analysis, involving comparison, reexamination, and grouping of codes. Descriptive themes were shared with and considered by all authors to ensure they were consistent and apposite. Descriptive themes were grouped and analytical themes were developed through an iterative process of reflection on, and interpretation of the descriptive themes within and across studies.

## Results

The search yielded 2909 records. Based on titles and abstracts, 168 records were selected for full text screening, resulting in a selection of 22 publications that met all eligibility criteria (Multimedia Appendix 2). Some of these studies were nested within larger trials of telehealth interventions. Multimedia Appendix 2 describes just the qualitative component extracted from each study.

**Figure 1:**
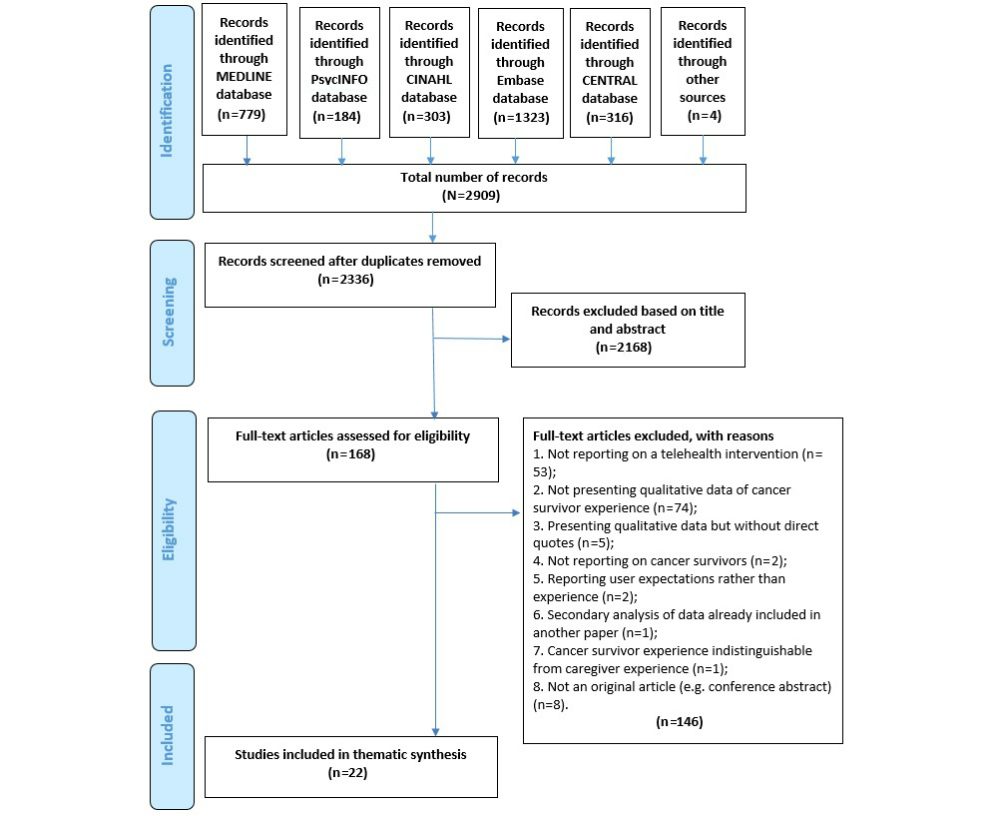
Preferred Reporting Items for Systematic Reviews and Meta-Analyses (PRISMA) study selection flowchart.

All the included studies were deemed to be of sufficient quality to contribute equally to the thematic synthesis. A Preferred Reporting Items for Systematic Reviews and Meta-Analyses (PRISMA) flowchart is presented in Figure 1.

### Characteristics of Included Studies

The 22 included studies used semistructured or in-depth interviews, or open-ended questions within surveys undertaken, with a total 445 patients (sample sizes listed in Multimedia Appendix 2). Of the total studies, 4 included only female survivors, [30-33], 1 study included only men [34], while the remaining 17 included both male and female participants [35-51]. Only 4 studies reported on the ethnicity of their participants [33,38,47,48]. Survivor cancer diagnoses included breast, lung, colorectal, ovarian, head and neck, prostate, hematological disease, and lymphoma. Study participants included adults who were newly diagnosed with cancer, those on active treatment, as well as those receiving follow-up care. The media used within telehealth interventions were heterogeneous: 11 studies appraised telephone-based interventions, [30-32,34,35,42,43,47,49-51], 5 studies related to primarily Web-based interventions, [33,36,39,40,48], 1 study evaluated email communication [37], and 5 looked at interventions using handheld monitoring devices [38,41,44-46]. The purpose of the interventions was diverse and included: 15 which supported patients through treatment; 6 which monitored symptoms [36,38,41,44-46]; and 9 which provided psychological support, information, advice or self-management strategies [33-35,39,40,42,43,47,49]. For 2 studies, telehealth acted as a form of communication between patients and HCPs at various stages of their journey [37,48] and 5 interventions replaced clinic visits for follow-up patients [30-32,50,51]. The majority of studies were carried out in the United Kingdom (Scotland and England; n=10). Others were conducted in the United States (n=3), Sweden (n=2), Canada (n=2), Australia (n=2), China (n=1) Hong Kong (n=1), and Singapore (n=1).

Regarding the aims of the 22 studies, 9 explored the views of patients and health care professionals as to the use of telehealth [30,41,44-48,50,51], 9 the experience of only patients [31,32,34-36,38,40,42,43], and 4 the experience of both patients and family members [33,37,39,49]. For 11 studies, the primary aim was to test the acceptability and feasibility of telehealth interventions [33-35,38,41-45,47,48]: 8 focused primarily on the experience of intervention use [30-32,40,46,49-51], and 3 aimed to explore the potential benefits of telehealth [36,37,39]. Of the 22 studies, 8 were qualitative studies nested within larger trials of telehealth interventions [30,34,38,40,46,47,49,51]. The intervention was nurse-led in 16 studies [30-32,34,36-41,43,45,47,49-51], with the remainder involving other health care professionals such as doctors or psychologists.

### Thematic Synthesis Findings

Three analytical themes encompassing patients’ experience of telehealth interventions emerged, with 3 descriptive themes underpinning each of these:

#### Theme 1: Influence of Telehealth on the Disrupted Lives of Cancer Survivors

This theme articulates how the remote nature of telehealth limited the disruption to peoples’ lives. Cancer survivors across many of the studies felt that their lives had been disrupted by the disease. Telehealth interventions enable the management of care remotely—away from the hospital environment—thereby minimizing this disruption. This analytical theme encompasses 3 descriptive themes: convenience, independence, and burden.

##### Convenience: “At My Convenience”

Results suggest there is benefit of telehealth in terms of convenience for cancer survivors, which allowed them to either return to normal activities or limit the interruption to daily routines. Ten studies reflected on this convenience in different ways. In telehealth interventions where telephone contact was used to replace face-to-face care, patients did not have to travel into the hospital, thereby saving time and money, and reducing the stress and burden of travel [30-32,50]. This level of convenience was highlighted as especially important for those with caring and work responsibilities [30,31,50]. Web-based or email-based interventions were viewed as flexible as they could be easily logged into at any time, meaning survivors could make contact or complete activities when it suited them and fitted into their lives [33,36,37,39,40,46]. Telehealth interventions were easily integrated into daily routines.

##### Independence: “I Learned What I Could Do”

Studies reviewed suggested that telehealth can alter the way in which survivors relate to HCPs. Remote consultations and monitoring mean survivors have a sense of physical independence, which puts an emphasis on self-care. Eight studies reported that participants felt telehealth had educated them about ways they could improve or manage their symptoms, or raised their awareness of potential issues to look out for with regards to their disease [36,38,41,43-45,51]. Survivors were given confidence to independently assess when they could manage their own care and when they should call for help [38,49]. In some cases, survivors reflected on their own data or summary information produced from the intervention, which motivated self-care efforts [45-47].

##### Burden: “Just One More Thing to Do”

If a telehealth intervention is difficult to engage with or time consuming, then it becomes a disruption in itself. Of the Web-based interventions, 2 were seen as an extra burden for survivors [36,48], while another study found that the online weekly supportive intervention was perceived by survivors as too time consuming [33]. In these studies, the possible convenience and independence that telehealth could provide was negated by these difficulties. “Burden” appears in contrast to “convenience.” These data suggest that survivors’ telehealth experiences are varied and complex; telehealth interventions may have “tipping points,” where they become burdensome instead of providing convenience.

#### Theme 2: Personalized Care Delivered From a Distance

This theme illustrates how telehealth can enable close and personalized relationships between cancer survivors and service providers even though the technology is remote and functions through physical distance. This perception of personalized care is underpinned by 3 descriptive themes: perception of personal space, which is created by survivors engaging with health care in their chosen environment, expanded sense of time that the remote environment engenders, and the effect remote connection has on the sense of human contact.

##### Space: “A Space I Was Missing”

In telehealth interventions, cancer survivors experience a different form of contact with their providers, engaging with their care physically from their place of choice. Of the included studies, 4 reported that remote communication gave them a sense of space to focus on their concerns and needs as they were in a familiar and relaxing environment [30,39,40,50]; while in another study where telephone follow-up replaced face-to face care, participants reported a sense that they were moving on and away from the hospital setting and its associations with disease [30]. The invisibility and perceived anonymity that telehealth provided reduced survivors’ sense of vulnerability, and in some cases enabled patients to raise concerns remotely that they would not have wanted to discuss face-to-face [40,47].

##### Time: “Time Was Never an Issue”

A prevalent theme reported across a number of studies (n=5) was that by being away from the clinical environment, survivors felt they had time to express their concerns and did not feel as rushed as they would have in a hospital setting [30-32,40,50]. Of the included papers, 3 focused on how communication with HCPs was enhanced due to these perceptions of increased available time [32,34,40]. In the 3 studies reporting on telehealth interventions using written communication, the medium seemed to defy the limits of time as the communication could be written or inputted at any point, and the response could be retained for future reference [37,39,40].

##### The Human Factor: “It Feels Impersonal”

Of the included studies, 9 reported that for some cancer survivors, telehealth was perceived as impersonal and lacking physical human contact [30,33,37,39,42,45,46,48,50], and 4 reported that survivors had not met in person the HCP they were connecting with via telehealth [30,39,42,50]. In some cases, a preference to know the HCP was related to the need for disclosure of personal and sensitive information [30,39].

The computer was highlighted as a particularly impersonal medium [33]; one study using a Web-based system reported that survivors were unsure whether their responses had been read by providers [48], and 3 other studies discussed how structured interventions were not tailored enough to survivors’ individual symptoms and concerns [45,46,48]. However, for other survivors, a structured format created a sense of security that all issues would be adequately considered [30,48,50].

#### Theme 3: Remote Reassurance—A Safety Net of HCP Connection

A common theme across the studies was that survivors felt they had immediate access to professional advice and that this acted as a safety net in that possible issues with their treatment, symptoms, or recovery would not be missed [30-32,35-37,40,41,43-46,49,51]. This was supported by 2 descriptive themes: where survivors could make an active connection with HCPs and where survivors felt passively monitored by providers. A third descriptive theme detailed instances when telehealth negatively affected the connection with HCPs.

##### Active Connection: “I Can Always Get in Touch”

Eight studies reported that telehealth interventions helped to reassure survivors by providing access to support and care through an active connection to HCPs [30-32,35,40,41,49]. Even if that opportunity was not used, cancer survivors felt a sense of safety knowing that they could make contact at any time [40]. In the case of telephone follow-up, patients valued the ease of being able to access a nurse between appointments, with rapid referral to the cancer service if needed [30,32]. This connection helped survivors feel safe; HCPs could offer reassurance at times of need or act swiftly to minimize any problems or concerns. For others, telehealth provided a sense of being cared for through the connection; somebody was at the end of the line to provide support [35,41].

##### Passive Connection: “Somebody to Keep an Eye on Them”

Eight studies identified how survivors felt monitored or watched over by health professionals in telehealth interventions [32,36,41,43-46,51], 5 studies reported on symptom management telehealth interventions where survivors entered data and HCPs responded when issues or problems arose [36,41,44-46], whereas 3 studies reported on interventions where survivors received phone calls from HCPs [32,43,51]. Contrary to the previous theme, the survivors in these interventions were not required to actively initiate contact with an HCP and instead were passively monitored. This perception of a “watchful eye” contributed to a sense of reassurance and ultimately to a sense that survivors were safe [45,46,51]. Patients felt that they were in the hands of a professional—an expert—who would be able to detect if survivors needed further tests, changes in medication or further intervention, and would set this in motion [32,40,43,44,51].

It is noteworthy that the reassurance provided by telehealth interventions was enhanced by the sense that telehealth provided consistency and continuity of contact. A trusting relationship, which extended “beyond the hospital boundaries” [40], was facilitated by this continuous contact [30,31,40,49]. While this aspect of cancer care may not be unique to telehealth, the data from these studies suggests that participants associate telehealth with continuity and consistency and contrast this with some impersonal clinical encounters [32].

##### Slipping Through the Net: “Missed the Connection”

The reassurance provided by the frequency and constancy of contact with HCPs in telehealth interventions was jeopardized in some studies [30,33,35,37,39,40,46,49,50]. In some cases, this arose when survivors allocated to telehealth were unable to engage with it due to particular personal circumstances, for example, survivors with hearing issues in a telephone-based intervention [35], or computer-based studies where survivors had poor computer literacy [37]. In 2 studies it was reported that technical issues in the telehealth intervention prevented connection being made [33,46]. In these examples, there was the sense that survivors’ concerns and issues might have slipped through the net.

Tables 1-3 list the studies reporting each of the above descriptive themes by their respective analytic themes. Table 4 provides a selection of quotes from participants to illustrate each theme.

**Table 1:** Themes identified in each study: influence of telehealth on the disrupted lives of cancer survivors.

Paper	Convenience “It didn’t really encroach”	Independence “I learned what I could do”	Burden “Just one more thing to do”
Beaver, Williamson, and Chalmers, 2010 [30]	✓^a^		
Chambers et al, 2015 [35]			
Chan et al, 2013 [36]	✓	✓	✓
Cornwall, Moore, and Plant, 2008 [37]	✓	✓	
Cox et al, 2008 [31]	✓		
Cox and Faithfull, 2015 [32]	✓		
Fergus et al, 2014 [33]	✓		✓
Hogberg et al, 2013 [39] Head et al, 2011 [38]	✓		
Hogberg et al, 2015 [40]	✓		
Kearney et al, 2006 [41]		✓	
Kilbourn et al, 2013 [42]			
Lai et al, 2015 [43]		✓	
Livingston et al, 2006 [34]			
Maguire et al, 2015 [44]		✓	
McCann et al, 2009 [46] McCall et al, 2008 [45]	✓	✓	
Ream et al, 2015 [47]		✓	
Snyder et al, 2013 [48]			✓
Stacey et al, 2016 [49]		✓	
Williamson, Chalmers, and Beaver, 2015 [50]	✓		
Zheng et al, 2013 [51]		✓	
			

^a^✓ indicates the theme was present within the paper.

**Table 2:** Themes identified in each study: personalized care delivered from a distance.

Paper	Space “A familiar and relaxing environment”	Time “Time was never an issue”	The human factor “It feels impersonal”
Beaver, Williamson, and Chalmers, 2010 [30]	✓^a^	✓	✓
Chambers et al, 2015 [35]			
Chan et al, 2013 [36]			
Cornwall, Moore, and Plant, 2008 [37]		✓	✓
Cox et al, 2008 [31]		✓	
Cox and Faithfull, 2015 [32]		✓	
Fergus et al, 2014 [33]			✓
Head et al, 2011 [38]			
Hogberg et al, 2013 [39]	✓	✓	✓
Hogberg et al, 2015 [40]	✓	✓	
Kearney et al, 2006 [41]			
Kilbourn et al, 2013 [42]			✓
Lai et al, 2015 [43]			
Livingston et al, 2006 [34]		✓	
Maguire et al, 2015 [44]			
McCall et al, 2008 [45]			✓
McCann et al, 2009 [46]			✓
Ream et al, 2015 [47]	✓		
Snyder et al, 2013 [48]			✓
Stacey et al, 2016 [49]			
Williamson, Chalmers, and Beaver, 2015 [50]	✓	✓	✓
Zheng et al, 2013 [51]			

^a^✓: indicates the theme was present within the paper.

**Table 3:** Themes identified in each study: remote reassurance-a safety net of health care professionals (HCPs) Connection.

Paper	Active connection “I can always get in touch”	Passive connection “Somebody to keep an eye on them”	Slipping through the net “Missed the connection”
Beaver, Williamson and Chalmers, 2010 [30]	✓^a^		✓
Chambers et al, 2015 [35]	✓		✓
Cornwall, Moore, and Plant, 2008 [37] Chan et al, 2013 [36]	✓		✓
Cox et al, 2008 [31]	✓		
Cox and Faithfull, 2015 [32]	✓	✓	
Fergus et al, 2014 [33]			✓
Head et al, 2011 [38]			
Hogberg et al, 2013 [39]			✓
Hogberg et al, 2015 [40]	✓	✓	✓
Kearney et al, 2006 [41]	✓	✓	
Kilbourn et al, 2013 [42]			
Lai et al, 2015 [43]		✓	
Livingston et al, 2006 [34]			
Maguire et al, 2015 [44]		✓	
McCall et al, 2008 [45]		✓	
McCann et al, 2009 [46]		✓	✓
Ream et al, 2015 [47]			
Snyder et al, 2013 [48]			
Stacey et al, 2016 [49]	✓		✓
Williamson, Chalmers, and Beaver, 2015 [50]			✓
Zheng et al, 2013 [51]		✓	

^a^✓ indicates the theme was present within the paper.

**Table 4:** Quotations from participants from primary studies to illustrate each theme.

Analytic themes	Descriptive themes	Quotations from participants in primary study
Influence of telehealth on the disrupted lives of cancer survivors	Convenience: “It didn’t really encroach”	“Because I’m still working, I’m self-employed and I travel all over the country... and it’s difficult sometimes to be at a hospital at a certain time. So that was good.” [30]
		“It was very easy, it was very simple to do and eh, it didn’t really encroach on lifestyle or anything like that at all, just had to remember to do it (laughs), set the ‘pinger’ on the cooker.” [46]
		“I haven’t got a car so I’d have to take two buses you see to go to the hospital. When I get to the hospital I have about an hour and a half wait in the waiting room. And I go see the doctor, 2 min and I'm out again.” [50]
	Independence: “I learned what I could do”	“It is educational in the sense that I have an overall view about the side effect of chemotherapy.” [36]
		“I learned what I could do to make myself feel better” [38]
		“I’m one of those people that likes statistics and numbers and things...and you could see on Tuesday I must have been quite bad...the graph’s right up and then it’s back down to normal today...” [45]
	Burden: “Just one more thing to do”	“We have very limited free time available and found it difficult to finish the lessons within a week.” [33]
		“It really was just one more thing to do. I didn’t feel that good a lot of the time so I really didn’t feel like doing one more thing. But I did it because I had to.” [48]
Personalized care delivered from a distance	Space: “A familiar and relaxing environment”	“It is much more relaxed to know that you don’t have the alien thing of the hospital. You can have it in your home (telephone follow-up). You have it at work. You can have it on your mobile if you want sat in the car.” [30]
	Time: “Time was never an issue”	“I felt that time was never an issue, that whatever I wanted to talk about, it was relevant. The time was given and it was discussed and that was good.” [32]
		“It has also been very nice with a written response. You can read it several times.” [40]
		“Quite happy. I did feel that I perhaps gleaned more information, I didn’t feel rushed or anything. And I’m sure that I sort of gleaned more information from my colorectal nurse than I would have perhaps done in a clinic situation.” [50]
	Human factor: “It feels impersonal”	“If I should share my innermost thoughts, I’d probably like to have some kind of relation with the person I’m writing to. Otherwise, I need to know exactly what I’m asking for.” [39]
		“But there was things I thought – noo, that’s how I feel and that’s what I’ve got but they’re no asking that, so I could’nae put it doon, do you know what I mean.” [46]
remote reassurance-a safety net of health care professionals (HCPs) Connection	Active connection: “I can always get in touch”	“The sessions helped me because there was somebody on the end of the line when you’re having a down day. And I mean if you’re having a down day you can ring them. You know it’s not like you’re alone in the world.” [35]
		“I used to make myself little cards that I carry round with me, one in my handbag, one at home here and one in my filing cabinet at work, so if I ever felt I needed to ring her up I’ve got ... ready access.” [32]
		“It’s really good to just have the opportunity lying there, I do not have to use it, but just knowing that there is a possibility is a security. That I can ask has been an incredible relief.” [40]
	Passive connection: “Somebody to keep an eye on them”	“It was quite positive. It was quite reassuring; you did feel that you were being monitored. You didn’t think if you put in those symptoms that you would slip through the...you know that if you had really worrying symptoms you would have slipped through the system. Somebody would have picked it up.” [46]
		“Well as far as I am concerned yes, because it was very helpful because I had this bad cough and 1 or 2 alerts came up and the nursing staff at the other end were immediately onto it the fact that we were in contact with the hospital very much quicker than we would be if we’d waited and maybe even phoned.” [44]
		“I found it helpful and interesting. It made me feel that my existence had some purpose...I think it is something which ought to be continued. It does make people feel they are being looked after...and somebody is keeping an eye on them.” [45]
		“I felt safe and reassured because the hospital staff followed up with me like a kite in their hand, so that I would not fly away.” [51]
	Slipping through the net: “Missed the connection”	“I have trouble on the phone, I have dreadful trouble with the mobile. Just mainly because of the complications with the hearing.” [35]
		“I got an answer that...made me...made me realize I had not put it (the issue) in the right way, and then I realized that I cannot sort this out, via this communication...with such long intervals.” [40]
		“I did miss the camaraderie that you get from other patients. And, of course, what tends to happen when you go on hospital visits is that you tend to be there at the same time as the other people who had their ops (operations) with you.” [50]

### Summary of Synthesis

The analytical constructs that have emerged through this qualitative synthesis demonstrate the complex experience of telehealth use in cancer survivorship. Telehealth can be experienced positively in terms of supporting a less disrupted life through providing convenience and independence to live life as a survivor rather than a patient. However, in order to embrace a more independent role, a trusted relationship with an HCP is crucial. This highlights an interplay for cancer survivors between appreciating the opportunity for home-based care and the reliance upon instant access to clinical support. Such interplay also exists between the convenience of such care and the increased responsibility, and potential burden, placed upon the survivor.

## Discussion

### Overview

**Figure 2:**
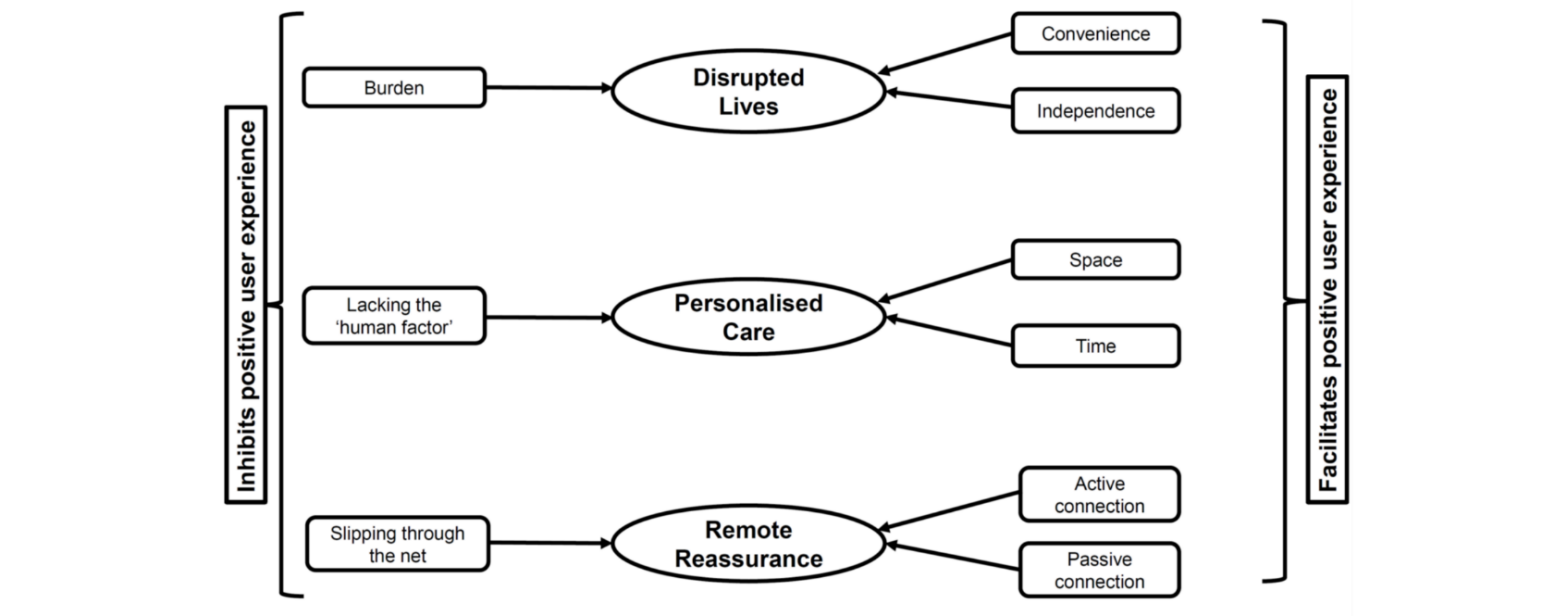
A model of cancer survivor engagement with telehealth—factors inhibiting and facilitating positive user experience.

This paper is the first to metasynthesize the reported experiences of cancer survivors who have participated in telehealth interventions. From the analysis, 3 key analytic themes and 9 descriptive subthemes emerged, showing that telehealth interventions in the area of cancer care represent a convenient approach, which can reduce treatment burden and disruption to cancer survivors’ lives. Our findings suggest that while telehealth interventions can facilitate an experience of personalized care for those living with and beyond cancer, interventions need to take personal factors into account so as to maximize benefit and minimize burden. The relationship between these themes is presented in a model (Figure 2) which summarizes our findings on cancer survivor experience of telehealth. Each of the analytical themes is presented in the center, and their descriptive themes on either side represent the factors inhibiting (left) or facilitating (right) the positive user experience of telehealth. These themes will be discussed below in the context of current literature.

### Principal Findings

The first analytic theme pertains to the concept of the cancer survivors’ disrupted life. Biographical disruption is a well-known consequence of chronic illness [52], and consistently in the literature cancer patients emphasize their desire to return to, and lead a life as “normal” as possible [53-59]. Yet there are a number of obstacles in the cancer survivor’s journey that can limit the ability to achieve this, particularly treatment burden [60]—an emerging concept within the chronic conditions literature including cancer [61]. Key sources of increased treatment burden for patients with chronic conditions include fragmented or poorly organized care lacking in continuity [62-65], poor communication with or between HCPs, barriers to accessing services, or insufficient time with health care professionals [63-65]. Our qualitative synthesis suggests that many of the above issues can in principle be addressed to some extent by telehealth provision, as demonstrated by the themes pertaining to time, convenience, and connection to HCPs. With treatment burden minimized and integrated into daily routines, biographical disruption from cancer survivorship becomes easier to address.

However, some cancer survivors experience telehealth as time-consuming [33] or as an additional burden [36,48], as reflected by one of the key issues facing telehealth provision: balancing benefit against burden. This issue has been highlighted in similar qualitative syntheses on telehealth interventions among patients with chronic conditions, for example, chronic obstructive pulmonary disease (COPD) [66]. To be successful, a telehealth intervention must balance any burden posed by technology and remote monitoring against the benefits of convenience and independence, as depicted in Figure 2. However, only 3 studies included in this review identified perceived burden resulting from the use of telehealth, and these consisted of trialing remote symptom reporting [36], patient-reported outcome completion [48], or Web-based coping or adjustment therapy [33], all requiring daily or weekly engagement with the intervention. This would suggest that while the majority of telehealth interventions included within our review were acceptable to cancer survivors in terms of the perceived balance of burden versus benefit, the required frequency of reporting or engaging with telehealth interventions is an important factor to consider in intervention design. Involvement of service users in the early stages of telehealth intervention design may be one way of ensuring this balance is maintained.

The second analytical theme represents the concept of personalized care from a distance. Enabling care within the home can offer benefits such as a familiar and relaxing environment within which to interact with an HCP, and the sense that the focus of care can shift toward the patient’s preferences and needs [67]. This is supported by this synthesis, as the feeling of having more time to communicate concerns was reported within 5 of the studies reviewed [30-32,40,50]. These results align with a recent metasynthesis identifying longer appointment times as being more accessible outside of the hospital care setting [68]. However, these advantages of telehealth are also accompanied by a certain feeling of remoteness, with survivors in some studies considering telehealth interventions (particularly computer or Web-based) to be impersonal or lacking in human contact, with patients feeling unsure whether anyone was “out there” listening to their submitted responses [48]. The issues of space, time, and impersonality are subsequently connected to personalized care in Figure 2.

This synthesis shows cancer survivors can experience telehealth interventions as lacking the “personal touch,” even when they are augmenting [33,37,39,42,45,46,48] rather than replacing [30,50] routine care. Nonetheless, some studies reviewed demonstrated that survivors were able to develop trusting relationships with HCPs via the telehealth medium [30,31,40,49], and other studies of telehealth interventions have demonstrated the capacity for such relationships to develop [69-71]. In addition, cancer survivors found they could more easily raise concerns with their HCP remotely, concerns that they would otherwise feel uncomfortable to discuss in person [40,47]. It can therefore be argued that personalized care, as enabled by telehealth interventions, can potentially provide reassurance and control to patients—that they can have the time and space to focus on articulating their health concerns.

This heterogeneity in the study findings pertaining to personalized care could be down to a number of factors, such as the method of delivering the intervention. For example, 4 of the 10 studies where patients reported a sense of impersonality in telehealth did not provide the opportunity to cancer survivors to meet their telehealth professional face-to-face prior to intervention delivery [30,39,42,50], despite face-to-face meeting being considered beneficial to promoting user engagement [72]. Another potential factor to consider is the population targeted within the included studies. In many of the studies where survivors were able to develop a trusting relationship with their HCP, the cancer survivors were relatively young, in their 20s and 30s (Multimedia Appendix 2). It could thus be argued that individuals who regularly use Web- or computer-based communication mediums may feel more comfortable with telehealth remote contact, and subsequently may find it easier to develop a relationship with their HCP. However, some concerns about ability to use technology in a telehealth context can be unfounded [73], and a case-by-case approach may be necessary to ensure that patients who struggle with technology can be provided some telehealth training so that they do not miss out. Overall, further exploration is required of the steps that need to be taken to encourage cancer survivors to develop a trusting relationship with telehealth care providers.

Further to the second analytic theme of “personalized care,” some survivors felt that telehealth interventions using structured symptom or patient-reported outcome questionnaires or providing self-care information were not sufficiently tailored to their circumstances [45,46,48], contributing to the sense that the intervention was impersonal. The advantage of a structured approach, for example, standard questionnaire items, is that it allows patients to know what symptoms they need to report. In the literature there are instances whereby chemotherapy symptoms were under-reported due to differences in self-care approach [74], or knowledge gaps in whether a symptom is due to cancer or the treatment received for the cancer [75,76]. However, some other studies included in our synthesis found that the structured format for logging responses was reassuring for survivors [30,48,50]. As findings are equivocal in this area, measures used in telehealth interventions may need to undergo a more iterative development process in order to increase personalization. This synthesis did not focus on the design process of telehealth interventions, but involvement of patients during this process, as discussed previously, could facilitate personalization. Ventura et al [77], in their evaluation of characteristics of eHealth supportive interventions (mainly in cancer), found that only 5 of 16 studies assessed had based intervention development on the needs assessment of the target population, indicating that consideration of individual needs at the early stages of telehealth development is still limited.

The final analytical theme identified was that of a “safety net” that cancer survivors felt was provided by either an active or passive connection to HCPs. Instances of active connection enabled the survivor to initiate the contact to receive support or advice, while passive connections such as responses to symptom or patient-reported outcome questionnaires, or routine telephone follow-up, were initiated by the HCP. This lead to survivors feeling reassured that they were being monitored, that medical assistance would be swift where it was deemed necessary, and that they could actively raise concerns. However, such connections may induce over-reliance on HCPs, potentially affecting cancer survivors’ autonomy and control. The risk for such dependency has been highlighted in recent reviews on telehealth in COPD [66] and chronic heart failure [78]. To date, there are no studies indicating the occurrence of any adverse events resulting from use of telehealth interventions in cancer care, therefore the dangers of this kind of dependency are unknown and represent an area for further evaluation. Given some instances highlighted in this review where survivors felt they may have “slipped through the net” due to technical problems [33,46] or not knowing whether their responses had been seen [48], ensuring consistency of monitoring during telehealth interventions is important, and steps can be taken to improve videoconferencing call quality and connection quality [79].

This synthesis indicates that telehealth interventions can provide cancer survivors with the necessary support they need to feel safe to manage their condition within their chosen environment. The findings can also be considered in terms of the person-based approach put forward by Yardley et al [80] for facilitating acceptance of eHealth interventions: promoting autonomy, competence (minimal disruption and achievement of self-regulation), and a positive experience of relatedness. Our findings suggest that the use of telehealth interventions with cancer survivors can facilitate autonomy and reduce disruption, and positive HCP relationships can be facilitated by remote monitoring. Thus, telehealth has the potential to address these needs. However, further research should address the personalization of telehealth, how to facilitate trusting survivor-HCP relationships, and how to mitigate the risks of dependency.

### Limitations

Only studies conducted since 2006 were included in this synthesis to capture the exponential increase in telehealth interventions over the past 10 years [83]. Therefore, the findings from our qualitative synthesis may not reflect cancer survivor experience of earlier telehealth interventions. Secondly, the ethnicity of participants was rarely reported—although studies contained a mix of cancer types, broad age ranges, and included all stages of disease from newly-diagnosed to the palliative care stages; people from black and minority ethnic groups may not have been adequately represented. Other demographic data such as languages spoken, health literacy level, presence or absence of cognitive impairment, and education level were also not reported in many studies, thus limiting our understanding of the experience of cancer survivors from underrepresented cultural and socio-economic groups. Similarly, due to the clinical heterogeneity of the samples included, it is not possible to draw conclusions regarding specific cancer types, disease stages, or age ranges that could benefit in particular from telehealth interventions.

The studies reviewed covered different disease stages, demonstrating that telehealth can support patients at any point in their cancer journey. It is noteworthy that overall the patients did not comment on the timing of the intervention, nor on its duration. Many of the survivors engaging in telehealth only do so for a relatively short period of time, with just 2 studies [30,32] engaging survivors with the telehealth intervention for 2 years or more. As a result, there was little data overall on the long-term experience of engagement with telehealth interventions for this group.

Arguably, other important factors might impact on cancer survivors’ experience of telehealth such as treatment stage, or the health care professional groups who are points of contact for the intervention. The conclusions drawn by this metasynthesis are limited by the research conducted to date which did not enable these factors to be addressed. Future research on telehealth interventions should explore the experience of cancer survivors at different stages of survivorship, and the impact of the HCPs monitoring these interventions on the experience of cancer survivors.

For 6 studies [31,33,36,37,41,49], qualitative data was collected only using open-ended survey questions, limiting the conclusions that could be drawn from survivor responses when compared with other studies which provided richer data. This synthesis only considered the experiences of adult cancer survivors who had participated in telehealth. Future reviews could also consider the experiences of HCPs, carers, or children and young adults, and the involvement of all these groups in the intervention design process. Research reporting the experiences of individuals who choose not to engage with telehealth or withdraw from interventions could also be explored to enhance understanding of the barriers to engagement in telehealth.

### Conclusions

This thematic synthesis supports the value of telehealth as a convenient and reassuring approach to delivering cancer care, which can minimize treatment burden and subsequent disruption to cancer survivors’ lives. As to how this synthesis could inform the development of future telehealth interventions, we would suggest that telehealth developers should balance the use of standardized patient outcomes measures with the introduction of more specifically tailored measures to minimize any sense of impersonal care. Furthermore, telehealth interventions need to be developed to balance benefit of remote monitoring and communication against burden, and consider survivor needs—perhaps through their involvement in the early stages of intervention design. The themes identified in the study are echoed in the existing literature on telehealth both in cancer and other long-term conditions. The model developed as part of this review therefore has the potential to not only facilitate understanding of the patient experience of telehealth in other conditions, but to guide the design of telehealth interventions in these areas to avoid factors that inhibit positive user experience, thereby improving telehealth engagement.

## Appendix

Multimedia Appendix 1 Search strategy for Medline.

Multimedia Appendix 2 Summary of characteristics of qualitative components of publications included in the review.

## Abbreviations

ANS: age not specified
ASyMS: advanced symptom management system
CNS: cancer not specified
CINAHL: Cumulative Index for Nursing and Allied Health Professionals
DNS: duration not specified
ENTREQ: Enhancing Transparency in Reporting the Synthesis of Qualitative Research
FNS: frequency not specified
GNS: gender not specified
HCP: health care professional
ND: newly diagnosed
