# Supplementary material for: Cancer Survivors’ Experience With Telehealth: A Systematic Review and Thematic Synthesis
Source: J Med Internet Res. 2017 Jan 9;19(1):e11. doi: 10.2196/jmir.6575 (PMC5259589; doi:10.2196/jmir.6575)
Supplement: Multimedia Appendix 1 [file jmir_v19i1e11_app1.pdf]

**Cancer survivors**	"Neoplasms"(MH) OR "Oncology Nursing"(MH) OR "Radiation Oncology"(MH) OR "Medical Oncology" (MH) OR "Oncology Service, Hospital" (MH) OR Cancer (TI) OR Oncology (TI)
**eHealth interventions**	"Management Information Systems"(MH) OR "Decision Making, Computer-Assisted" (MH) OR "Therapy, Computer-Assisted" (MH) OR "Medical Records Systems, Computerized" (MH) OR “Technology” (MH) OR "Remote Sensing Technology" (MH) OR "Technology Assessment, Biomedical" (MH) OR "Telecommunications"(MH) OR "Telephone" (MH) OR "Cell Phones" (MH) OR "Electronic Mail" (MH) OR "Videoconferencing" (MH) OR "Telemedicine" (MH) OR "Telenursing" (MH) OR "Computers, Handheld" (MH) OR "Mobile Applications" (MH) OR "Remote Consultation" (MH) OR "Computer Communication Networks" (MH) OR "Attitude to Computers" (MH) OR "Internet" (MH) OR App (TI) OR Apps (TI) OR Device* (TI) OR M-health (TI) OR Mhealth (TI) OR Mobile* (TI) OR Phone* (TI) OR Smartphone* (TI) OR Telephone* (TI) OR E-health (TI) OR Ehealth (TI) OR Web* (TI) OR On-line (TI) OR Online (TI) OR Smartphone (TI)
**Survivor experience**	"Patient Acceptance of Healthcare" (MH) OR "Patient Satisfaction" (MH) OR "Patient-Centered Care" (MH) OR Experience* (TI) OR Accept* (TI) OR Satisf* (TI) OR Perception* (TI) OR Perspective* (TI) OR View* (TI) OR Attitude* (TI) OR ((Patient* or User*) AND (Experience* OR Accept* OR Satisf* OR Perception* OR Perspective* OR View* OR Attitude*)) (ABS)
**Combination**	Cancer survivors AND eHealth interventions AND survivor experience
**Limiters**	1) English language only<br>2) Last 10 years (1/1/2006 – 31/8/2015)<br>3) Adult only<br>4) Human
