# Supplementary material for: Cancer Survivors’ Experience With Telehealth: A Systematic Review and Thematic Synthesis
Source: J Med Internet Res. 2017 Jan 9;19(1):e11. doi: 10.2196/jmir.6575 (PMC5259589; doi:10.2196/jmir.6575)
Supplement: Multimedia Appendix 2 [file jmir_v19i1e11_app2.pdf]

Multimedia Appendix 2. Summary of characteristics of qualitative components of publications included in the review.

| Study | Primary aim | Survivor demographics (gender cancer type age) | Telehealth intervention (type purpose frequency duration) | Telehealth role | Data collection method (Analysis method) |
|---|---|---|---|---|---|
| Beaver, Williamson, and Chalmers, 2010 England, United Kingdom | Patient and HCP[a] views on telephone follow-up posttreatment for breast cancer. | 28F[b] breast cancer 48-80 | Telephone Patient information or support 30 minutes (FNS[c]) DNS[d] | Replace face-to-face follow-up care | Interviews (Content analysis) |
| Chambers et al, 2015 Australia | Acceptability of telephone-delivered psychological wellness intervention. | 13 (GNS[e]) lung cancer 65 | Telephone Cognitive behavioral therapy 55 minutes/week 6 weeks | Remote patient support | Interviews (Thematic analysis) |
| Chan et al, 2013 Singapore | Evaluate online home monitoring + symptom management of chemotherapy patients. | 3F, 1M breast cancer colorectal cancer ND[f] 39-59 | Web-based Symptom reporting + self-care + videoconferencing Daily symptom reports, 2 x video conference assessments 4 chemotherapy cycles | Symptom management | 1 open-ended survey question (Thematic analysis) |
| Cornwall, Moore, and Plant, 2008 England, United Kingdom | Evaluate email communication method between nurses, patients, and carers. | 2M[g], 2F lung cancer 33-77 | Email Communication method As and when desired by all parties 6 months | Communication tool | Open-ended survey questions (Not specified) |

| Study | Primary aim | Survivor demographics (gender cancer type age) | Telehealth intervention (type purpose frequency duration) | Telehealth role | Data collection method (Analysis method) |
|---|---|---|---|---|---|
| Cox et al, 2008, England, United Kingdom | Evaluate nurse led telephone intervention to encourage proactive ovarian cancer management, including physical + psychological morbidity. | 46F<br>ovarian cancer stage II to IV<br>18 < 60<br>28 ≥ 60 | Telephone<br>Check symptoms/ recurrence + self-care advice<br>20 minutes every 3 months or when patient desires<br>DNS | Replacement of face-to-face follow-up care | Open-ended survey questions (Content analysis) |
| Cox and Faithfull, 2015, England, United Kingdom | Explore views/ experiences of women receiving long-term (≥ 3 years) nurse-led telephone follow-up. | 11F<br>ovarian cancer<br>47-79 | Telephone<br>Check symptoms/ recurrence + self-care advice<br>20 minutes every 3 months or sooner if treatment demanded<br>>3 years | Replacement of face-to-face follow-up care | Interviews (Interpretative phenomeno-logical analysis) |
| Fergus et al, 2014, Canada | Feasibility + acceptability of online intervention to support young couples' coping and adjustment to breast cancer. | 10F<br>breast cancer<br>33 | Web-based discussion board + telephone<br>online coping/ adjustment intervention + 2 telephone calls<br>Weekly<br>8 weeks | Remote patient support | Survey with open-nded questions and interview (Content analysis) |
| Head et al, 2011, United States | Feasibility + acceptability of symptom management telehealth intervention during treatment for head + neck cancer. | 5F, 39M<br>head & neck cancer<br>59 | Device: Health Buddy<br>Education/ support + symptom management + self-care<br>Daily<br>Throughout treatment and 2 weeks posttreatment) | Symptom management | Interviews (Thematic analysis) |

| Study | Primary aim | Survivor demographics (gender cancer type age) | Telehealth intervention (type purpose frequency duration) | Telehealth role | Data collection method (Analysis method) |
|---|---|---|---|---|---|
| Högberg et al, 2013, Sweden | Describe patient/family perspective of Web-based communication tool for psychosocial support. | 5F, 6M hematological cancer any stage 22-68 | Web-based Patient information or support As patient desires with nurse response within 3 days DNS | Remote patient support | Interviews (Content analysis) |
| Högberg et al, 2015, Sweden | To describe the meaning of using Web-based communication for support from a patient perspective. | 6F, 4M hematological cancer any stage 21-72 | Web-based Patient information or support As patient desires with nurse response within 3 days DNS | Remote patient support | Interviews (Hermeneutic approach) |
| Kearney et al, 2006, Scotland, United Kingdom | Patient/HCP perceptions on ease/benefits of using a symptom management handset. | 5F, 10M lung cancer colorectal cancer 24-77 | Device: Handheld Computer Realtime symptom-reporting + self-care Daily reporting, DNS | Symptom management | Survey with open-ended question (Thematic analysis) |
| Kilbourn et al, 2013, United States | Feasibility, acceptability + benefits of telephone-based psychosocial intervention to improve symptom management. | 8 (GNS) head & neck cancer ND 59 | Telephone-based Symptom management + needs assessment + self-care Max 8 sessions aligned with time of diagnosis + active treatment + end of treatment | Remote patient support | Interviews (Thematic analysis) |
| Lai et al, 2015, Hong Kong | Feasibility + acceptability of nurse-led care program for chemotherapy patients. | 4F, 1M breast cancer colorectal cancer 42-69 | Telephone Assessment + triage + self-care 5-24 minutes, After 1$^{st}$ + 2$^{nd}$ chemotherapy cycles | Remote patient support | Interviews (Content analysis) |

| Study | Primary aim | Survivor demographics (gender cancer type age) | Telehealth intervention (type purpose frequency duration) | Telehealth role | Data collection method (Analysis method) |
|---|---|---|---|---|---|
| Livingston et al, 2006, Australia | Feasibility + acceptability of telephone cancer information support service. | 81M colorectal cancer prostate cancer stages 1–3 ND ANS[h] | Telephone Discuss cancer diagnosis + treatment + management issues Randomized to 4 calls; 1 call with patient left to call back if they wish; or patient required to call 6 months | Remote patient support | Interviews (Thematic analysis) |
| Maguire et al, 2015, United Kingdom | Feasibility + acceptability of ASyMS[i] in lung cancer patients receiving radiotherapy + clinicians providing care. | 10 (GNS) lung cancer 42-85 | Device: ASyMS Realtime symptom-reporting + self-care Daily reporting Duration of radiotherapy + 1 month posttreatment) | Symptom management | Interviews (Thematic analysis) |
| McCall et al, 2008, Scotland, United Kingdom | Acceptability + usability of ASyMS in palliative care. | 2F, 4M CNS[j] late stage cancer aged 40-87 | Device: ASyMS Realtime symptom-reporting + self-care Daily reporting 30 days | Symptom management | Interviews & survey with open-ended question (Thematic analysis) |
| McCann et al, 2009, Scotland, United Kingdom | Patient and HCP perspectives/ experiences of using ASyMS. | 12 (GNS) breast cancer lung cancer colorectal cancer ANS | Device: ASyMS Realtime symptom-reporting + self-care Twice-daily reporting x 14 days, Four chemotherapy cycles | Symptom management | Interviews (Thematic analysis) |

| Study | Primary aim | Survivor demographics (gender cancer type age) | Telehealth intervention (type purpose frequency duration) | Telehealth role | Data collection method (Analysis method) |
|---|---|---|---|---|---|
| Ream et al, 2015, United Kingdom | Explore patients' perceptions of feasibility + acceptability of a fatigue management telephone intervention. | 9 (GNS)<br>breast cancer<br>colorectal cancer<br>lymphoma<br>ANS | Telephone<br>Fatigue self-care + activities,<br>3 calls, 20-40 minutes<br>3 treatment cycles | Remote patient support | Interviews<br>(Not specified) |
| Snyder et al, 2013, United States | Feasibility, value, usefulness + acceptability of Patient Viewpoint website for patients and clinicians. | 34F, 13M<br>breast cancer<br>prostate cancer<br>any stage<br>28-81 | Web-based<br>PRO collection<br>Every 2 weeks<br>DNS | Communication tool | Interviews<br>(Not specified) |
| Stacey et al, 2016, Canada | Patient + family experiences with telephone cancer treatment symptom support. | 43 (GNS)<br>CNS<br>25-83 | Telephone<br>Symptom management + advice<br>As and when patient desires<br>DNS | Remote patient support | Survey with open-ended question<br>(Content analysis) |
| Williamson, Chalmers, and Beaver, 2015, United Kingdom | Patient experience of telephone follow-up for colorectal cancer. | 9F, 12M<br>colorectal cancer<br>6 < 60<br>15 ≥ 60 | Telephone<br>Patient information or support<br>FNS<br>DNS | Replacement of face-to-face follow-up care | Interviews<br>(Content analysis) |
| Zheng et al, 2013, China | Explore patient + nurse views of telephone follow up postcolostomy | 4F, 7M<br>colorectal cancer,<br>28-71 | Telephone<br>Discuss assessment + management<br>2-3 telephone calls within 1 month of hospital discharge<br>DNS | Replacement of face-to-face follow-up care | Interviews<br>(Content analysis) |

[a]HCP: health care professional.

[b]F: Female.[c]FNS: frequency not specified.

[d]DNS: duration not specified.

[e]GNS: gender not specified.

[f]ND: newly diagnosed.

[g]M: Male.

[h]ANS: age not specified.

[i]ASyMS: Advanced Symptom Management System.

[j]CNS: cancer no specified.
